# Impact of a small-scale tsetse fly control operation with deltamethrin impregnated “Tiny Targets” on tsetse density and trypanosomes’ circulation in the Campo sleeping sickness focus of South Cameroon

**DOI:** 10.1371/journal.pntd.0011802

**Published:** 2023-11-27

**Authors:** Tito Tresor Melachio Tanekou, Calmes Ursain Bouaka Tsakeng, Inaki Tirados, Alphonse Acho, Jude Bigoga, Charles Sinclair Wondji, Flobert Njiokou

**Affiliations:** 1 Centre for Research in Infectious Diseases (CRID), Yaoundé, Cameroon; 2 Department of Microbiology and Parasitology, Faculty of Science, University of Bamenda, Bamenda, Cameroon; 3 Department of Vector Biology, Liverpool School of Tropical Medicine, Pembroke Place, Liverpool, United Kingdom; 4 Department of Biochemistry, Faculty of Science, University of Yaoundé I, Yaoundé, Cameroon; 5 Programme National de Lutte contre la Trypanosomose Humaine Africaine (PNLTHA), Ministère de la Santé Publique, Cameroon; 6 Laboratory for Vector Biology and Control, National Reference Unit for Vector Control, The Biotechnology Centre, Nkolbisson, Yaoundé, Cameroon; 7 Department of Animal Biology and Physiology, Faculty of Science, University of Yaoundé I, Yaoundé, Cameroon; University of Notre Dame, UNITED STATES

## Abstract

**Background:**

Significant progress has been made towards African sleeping sickness elimination in the last decade. Indeed, the World Health Organization (WHO) global goal of eliminating the chronic form of the disease as a public health problem was achieved in 2020 (i.e., < 2,000 new cases per year). Vector control has played an important role in achieving this goal. In this study, we evaluated the impact of the insecticide impregnated Tiny Targets on tsetse fly densities and their infection rates with *Trypanosoma* spp in the Campo sleeping sickness focus of South Cameroon.

**Methods:**

The study site was divided into two areas: (i) the south-west experimental area, which included vector control, and (ii) the eastern part as the non-intervention area. After compiling the baseline entomological data (tsetse densities and trypanosome infection rates), around 2000 Tiny Targets were deployed in the South-West area and replaced every six months for two years. Post-intervention surveys were conducted every six months to determine tsetse densities and levels of trypanosome infections with PCR-based methods.

**Results:**

Following the intervention, tsetse mean catches decreased by 61% after six months, and up to 73% after twelve months (pre-intervention: 2.48 flies/trap/day, 95%CI [1.92–3.14]; 12-months post-intervention: 0.66 tsetse/trap/day, 95%CI [0.42–0.94]). This decrease was not sustained after 18 months, and the mean catch doubled compared to that after 12 months. After 24 months, the mean catches still increased by 17% (18 months: 1.45 tsetse/trap/day, 95%CI [1.07–1.90] and 24 months: 1.71 tsetse/trap/day, 95%CI [1.27–2.24]). In the non-intervention area, a variation in tsetse catches was observed during the two years, with a general increase from 2.43 [0.73–5.77] to 3.64 [1.47–7.70] tsetse/trap/day. In addition, trypanosome infection rates dropped by 75% in both areas (P-value < 0.001) from 21.20% to 5.06% and from 13.14% to 3.45% in intervention and control areas respectively.

**Conclusion:**

Tiny targets have proven useful in reducing tsetse population densities and trypanosome infection rates, providing evidence for the integration of this tool in current strategies towards trypanosomiasis elimination in Campo. The non-sustained decrease of tsetse densities after one year may indicate reinvasions from neighbouring breeding sites or that the intervention area was not large enough. Our results show the need to scale up by accessing difficult breeding sites and extend the tiny targets to the whole transborder focus.

## Author summary

Human sleeping sickness caused by trypanosomes still affects the poor community of Campo in the South Region of Cameroon. During routine activities of farming, fishing, and hunting in the forest, villagers are in close contact with tsetse flies which bite them and transmit the disease. The tsetse fly display high densities in Campo, increasing the risk of disease transmission to the population. Recently, deltamethrin-treated blue-black fabric screens called Tiny Targets were developed to reduce tsetse densities when installed in the environment. These Tiny Targets significantly contributed to decrease sleeping sickness cases in many countries. In this study, baseline data were collected on tsetse densities in Campo and after population sensitization, around 2000 Tiny Targets were installed and maintained for two years with help of 10 villagers who received prior training. The Tiny Targets were replaced every six months and monitoring activities showed that tsetse density decreased by 73% after one year with a slight recovery in the second year. Trypanosome infections in tsetse flies also decreased by 75%. This study showed the positive impact of Tiny Targets in Campo in limiting trypanosome circulation and suggested to scale up the area covered by the tsetse control, to improve its efficiency.

## Introduction

Trypanosomiases are infectious diseases affecting humans and animals. They are caused by several *Trypanosoma* species, cyclically transmitted by tsetse flies of the genus *Glossina*. Human African Trypanosomiasis (HAT), also known as sleeping sickness, is an important but neglected public health disease in sub-Saharan Africa [1]. In West and Central Africa, *Trypanosoma brucei gambiense* causes the chronic form of HAT, responsible for >98% of all officially reported HAT cases, while in East and Southern Africa, *Trypanosoma brucei rhodesiense* causes the acute form of the disease which is responsible for <2% of reported cases [2]. Animal African Trypanosomiasis (AAT), also known as “nagana”, is a major constraint to livestock production in 37 sub-Saharan African countries [3,4]. About 50 million cattle are at risk of AAT with an estimated economic cost of US$4.5 billion per year [5] and therefore the disease is a cause of poverty and famine.

Great progress has been made towards the elimination of sleeping sickness throughout endemic areas in Africa. WHO reports of 1998 highlighted that about 300,000 new cases of HAT occurred every year [6]. As a result of great efforts deployed by WHO, governments and other stakeholders to improve human case detection & treatment and vector control, the number of new cases recorded declined to 50,000–70,000 by 2006 [7]. Over the past two decades, the development and implementation of cost effective vector control [8–11] have helped to further decrease the HAT incidence, with the lowest records of 2164 new cases in 2016 [12], 977 in 2018 [13] and 565 in 2020 [14]. After achieving the global goal of eliminating the *gambiense* HAT (gHAT) as a public health problem in 2020, WHO and partners were highly encouraged to move towards the goal of complete interruption of transmission of gHAT by 2030.

In Cameroon, the disease mainly occurs in Campo with more than 90% of the cases in the country being reported from this area. Campo is a transborder focus located in the South Forest Region and shared between Cameroon and Equatorial Guinea. Around seven new cases occurred each year between 2012 and 2018 in the Cameroonian part of the focus [14]. In 2019 however, the number of reported new cases increased drastically to 20 [15]. Recent studies in Campo have shown that the tsetse vectors of *g*HAT display high densities, thus maintaining the risk of transmission of both human and animal trypanosomes at high levels [16–19].

The insecticide impregnated Tiny Targets have recently proven efficient for tsetse control and accelerating elimination of gHAT in African foci [8,20]. In the present study, we introduced Tiny Targets for the first time in Campo at a small scale to assess their efficiency in a forest/mangrove context. This study was a pilot study conducted in the frame of the PIIVeC Project (Partnership for Increasing the Impact of Vector Control - https://www.piivec.org/ -), which aims to improve the policies of fighting vector-borne diseases through the identification and implementation of appropriate actions to control their vectors.

## Material and methods

### Study area

**Fig 1:**
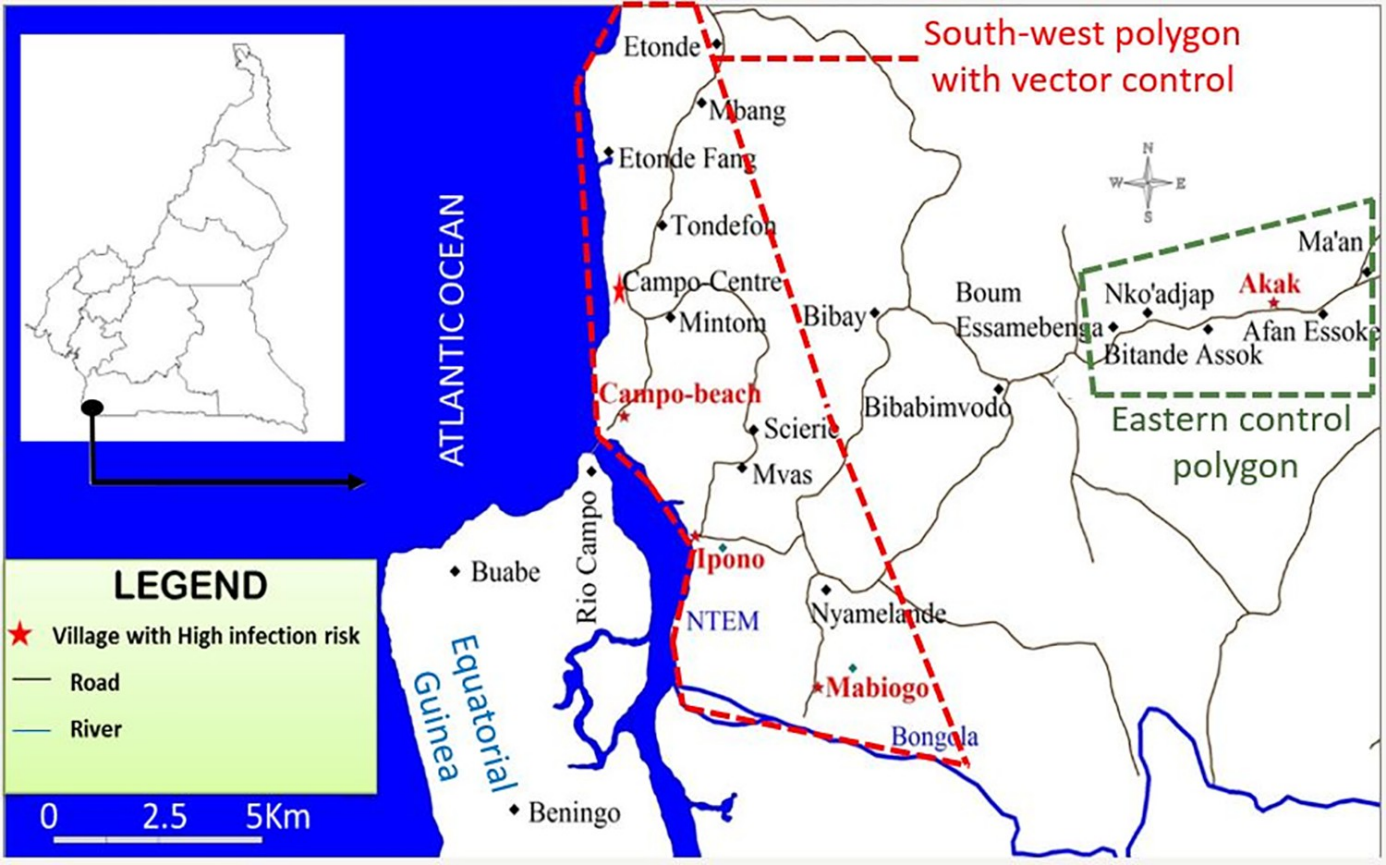
Study area—Campo, South Cameroun. Figure designed using the ESRI satellite base map (https://www.arcgis.com/home/webmap/viewer.html?url=https%3A%2F%2Fserver.arcgisonline.com%2Farcgis%2Frest%2Fservices%2FWorld_Imagery%2FMapServer&source=sd).

Campo (2° 22’ N; 9° 49’ E) is in the South region of Cameroon, on the western Atlantic coast and borders Equatorial Guinea in the South. Campo extends along the Ntem river and is characterised by typical maritime equatorial climate with four seasons of approximately 3 months each. There are two rainy seasons and two dry seasons of inequal characteristics, i.e., the heavy dry season is characterised by relatively lower rain levels and higher temperatures than the light dry season. The environment is composed of a dense hydrographic network with several rivers, a coastal plain along the ocean, a mangrove swamp along the Ntem River in the southern part of the area, marshes, and evergreen forest. The main activities of Campo inhabitants are fishing, farming, and hunting. However, hunting has been highly restricted since the creation of Campo/Maan national reserve park where wild fauna composition is highly diverse [21]. Across the River Ntem, significant population movements are observed between Campo Beach (Cameroon) and Rio Campo (Equatorial Guinea) for economic and familial purposes. These exchanges of populations from both sides may impact the epidemiology of several diseases [22], including HAT. The study area maps were designed under the software QGIS [23], using the ESRI satellite base map (Environmental Systems Research Institute, Inc., Redlands, CA, www.esri.com) that is publicly available. The area was divided into two polygons (Fig 1): The South-West polygon–where HAT cases have been reported for decades–was chosen as intervention area, where vector control was implemented. The Eastern polygon, located around 10 km from the intervention area, was used as the non-intervention area with no Tiny Targets deployed.

### Tsetse pre-intervention surveys

Two baseline entomological surveys were conducted in December 2018 and July 2019 (heavy and light dry seasons respectively) for pre-intervention data collection. Tsetse flies were collected using pyramidal traps [24] placed in suitable biotopes (water points, riverbanks, behind dwellings, along the roads, farmlands). The trap positions were geo-referenced using a global positioning system. The trapping points were chosen following a similar preliminary work done in the same area in 2012 [18], so that the bulk of the area was covered. The traps were visited once a day for 2 to 4 consecutive days, and in each visit, tsetse trapped were collected and transferred into the field laboratory. Once in the field laboratory, a morphological identification of the tsetse flies was carried out to determine their species, sex and their teneral/non-teneral status (i.e., if they had taken their first blood meal or not). Tsetse flies were then placed individually in Eppendorf tubes containing ethanol 95%. Once in the main laboratory, the tubes were stored at -20°C until subsequent analyses.

### Population sensitization, tiny targets deployment and maintenance

**Fig 2:**
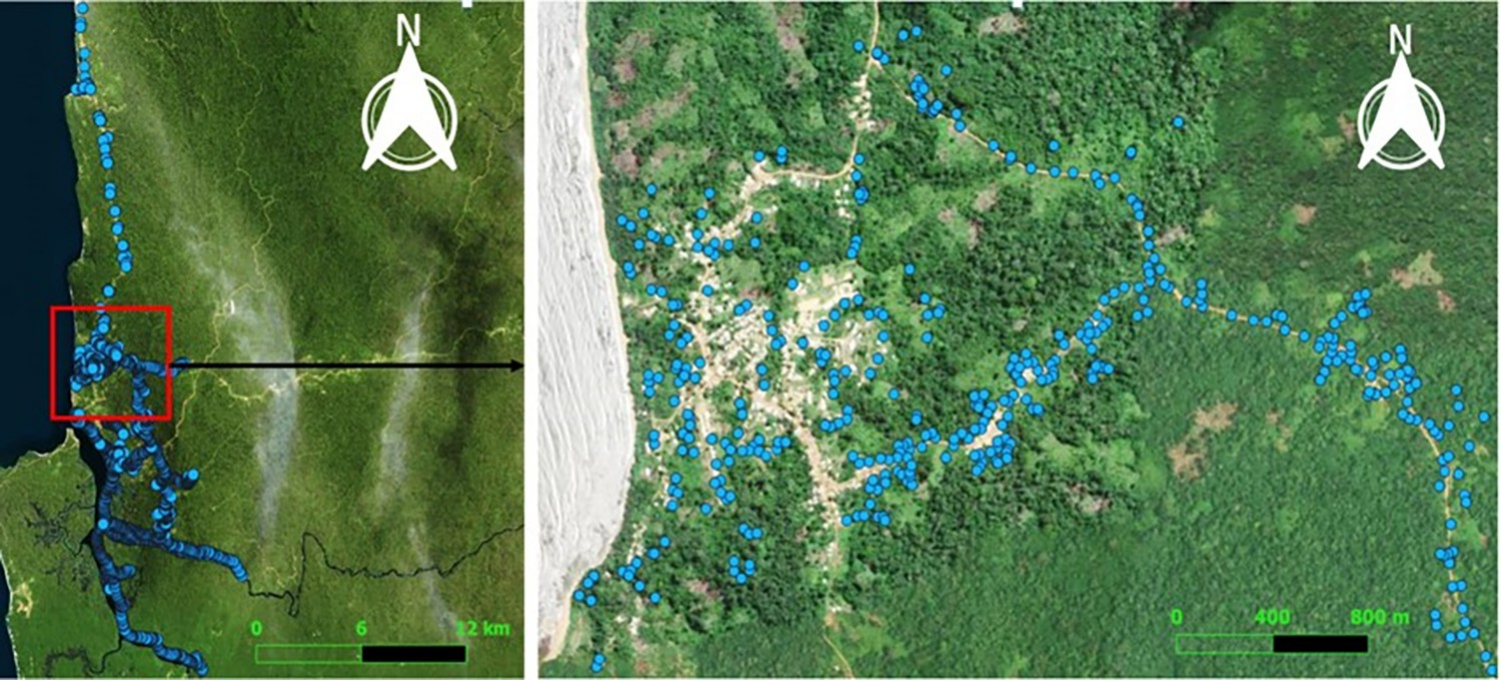
Location and distribution of tiny targets in the intervention area. Figure designed using the ESRI satellite base map (https://www.arcgis.com/home/webmap/viewer.html?url=https%3A%2F%2Fserver.arcgisonline.com%2Farcgis%2Frest%2Fservices%2FWorld_Imagery%2FMapServer&source=sd).

Prior to the deployment of tiny targets in January 2020, a preliminary sensitization campaign was carried out through a meeting organized with local authorities and the population in the village Campo Beach. This was followed by a five-days door-to-door sensitization to explain the project activities, prepare the population about the upcoming installation of Tiny Targets in their environment, and their participation or engagement to help improve the performance of the tool. Approximately 2000 tiny targets (Vestergaard-Frandsen, Lausanne, Switzerland) were then deployed in January 2020 (Fig 2) and replaced every six months to cover a period of 2 years (August 2020, January 2021, and August 2021). The deployments were conducted during the dry season. This season allows easy access to the fly breeding sites and is favourable for the flies’ biological activities (feeding and reproduction), thus facilitating their contact with the screens. The locations of all targets were recorded using global positioning system units and mapped using the software QGIS 3.16. The installation was carried out by the research team in association with local people. Local assistants received prior training on how to mount and set up the targets, and how to record their locations. Targets were installed at intervals of approximately 50m. The vegetation surrounding the targets was cleared in a radius of 1.5m, so that they could be easily visible for the flies. During redeployments, we minimised the impact on the environment by removing old Tiny Targets for further destruction under controlled conditions.

### Tsetse post-intervention surveys

Entomological monitoring was conducted at 90 geo-referenced sentinel positions (77 in the intervention area and 13 in the non-intervention control area), which were the same used for baseline entomological data collection. The same sites were used before the first deployment (to provide the baseline data on mean catches) and after the interventions (to provide a measure of the intervention impact). These surveys were carried out every six months before the replacement of Tiny Targets (August 2020, January 2021, August 2021) and in January 2022 at the end of the vector control operation. Collected flies were identified (species and sex), counted, and stored in 95% ethanol for subsequent laboratory-based analyses (e.g., identification of trypanosome infections).

### DNA extraction and identification of trypanosome infections in tsetse flies

Tsetse heads (mouthparts) were separated from the rest of the bodies to assess mature infections to trypanosomes. Total DNA was extracted from tsetse fly bodies using the LIVAK protocol [25], and *Trypanosoma* species were detected by a nested PCR as described by Desquesnes *et al*. [26], amplifying the Internal transcribed spacer 1 of the rDNA. The primer pairs used were TRYP18.2C (5’-GCAAATTGCCCAATGTCG-3’) & TRYP4R (5’-GCTGCGTTCTTCAACGAA-3’) for the first round, and IRFCC (5’-CCTGCAGCTGGATCAT-3’) & TRYP5RCG (5’-ATCGCGACACCTTGTG-3’) for second reaction. From flies found with trypanosome DNA in their bodies, DNA was extracted from mouthparts and tested for presence of trypanosomes using the same protocols described for trypanosome detection in bodies.

### Statistical analyses

Tsetse fly apparent densities per trap (ADT) were calculated as the total number of flies captured divided by the number of traps used and by the number of days of captures. Mean ADTs were computed after normalization of mean daily tsetse catches per trap (e.g., $y_{i}=\log(x_{i}+1)$), followed by computing the arithmetic mean of the transformed catches and back-transformation of the mean using the equation: $ADT=10^{\bar{y}}-1$. The mean catches (ADTs) were reported along with their 95% confidence intervals. ADTs between different monitoring periods were compared through analysis of variance using Graphpad Prism8 (GraphPad Software, Boston, Massachusetts USA, www.graphpad.com).

Trypanosome infection rates in tsetse were computed as the percentage of flies found with trypanosome DNA over the total number of flies analysed. A Chi-square test was used to compare the infection rates of different trypanosome species between sampling periods, and before and after vector control with tiny targets, using the software R [27] with the significance threshold set at 0.05.

## Results

### Impact of tiny targets on tsetse densities

#### Variation of tsetse densities in the intervention area

Out of the 2287 tsetse flies that were caught during the pre-intervention surveys in the intervention area, 1312 (57.37%) and 975 (42.63%) were from heavy (December 2018) and light (July 2019) dry seasons respectively. Four tsetse fly species or sub-species were identified, namely *Glossina palpalis palpalis*, *G*. *pallicera*, *G*. *caliginea* and *G*. *nigrofusca* (Table 1). *G*. *palpalis palpalis*, which is the sub-species responsible for the transmission of the human sleeping sickness in Campo, was largely dominant, with overall relative abundance of 92.44% and 93.23% for the two seasons, respectively. This species also displayed high densities in the different sampling points, with mean ADT values of 2.15 95%CI [1.60–2.81], and 2.48, 95%CI [1.92–3.14] flies/trap/day in heavy and light dry seasons respectively.

**Table 1:** Summary of the number of flies captured before and during tsetse control in the intervention area.

Tsetse sp	*G*. *palpalis palpalis*	*G*. *caliginea*	*G*. *nigrofusca*	*G*. *pallicera*	Non identified	Total	*G*.*p*.*p*. ADT
**December 2018**	1218 (92.84)	38 (2.90)	2 (0.15)	53 (4.04)	1 (0.08)	1312	2.15 [1.60–2.81]
**July** **2019**	909 (93.23)	12 (1.23)	6 (0.62)	47 (4.82)	1 (0.10)	975	2.48 [1.92–3.14]
**August** **2020**	471 (97.72)	7 (1.45)	0 (0)	4 (0.83)	0 (0)	482	0.95 [0.69–1.35]
**January** **2021**	403 (95.27)	12 (2.84)	0 (0)	8 (1.89)	0 (0)	423	0.66 [0.42–0.94
**August** **2021**	572 (96.79)	1 (0.17)	5 (0.85)	12 (2.03)	0 (0)	591	1.45 [1.07–1.90]
**January** **2022**	662 (91.18)	14 (1.93)	1 (0.14)	29 (3.99)	0 (0)	726	1.71 [1.27–2.24]

*G*.*p*.*p*.: *Glossina palpalis palpalis*; Numbers in brackets are relative abundances in percentage of each tsetse taxon.

**Fig 3:**
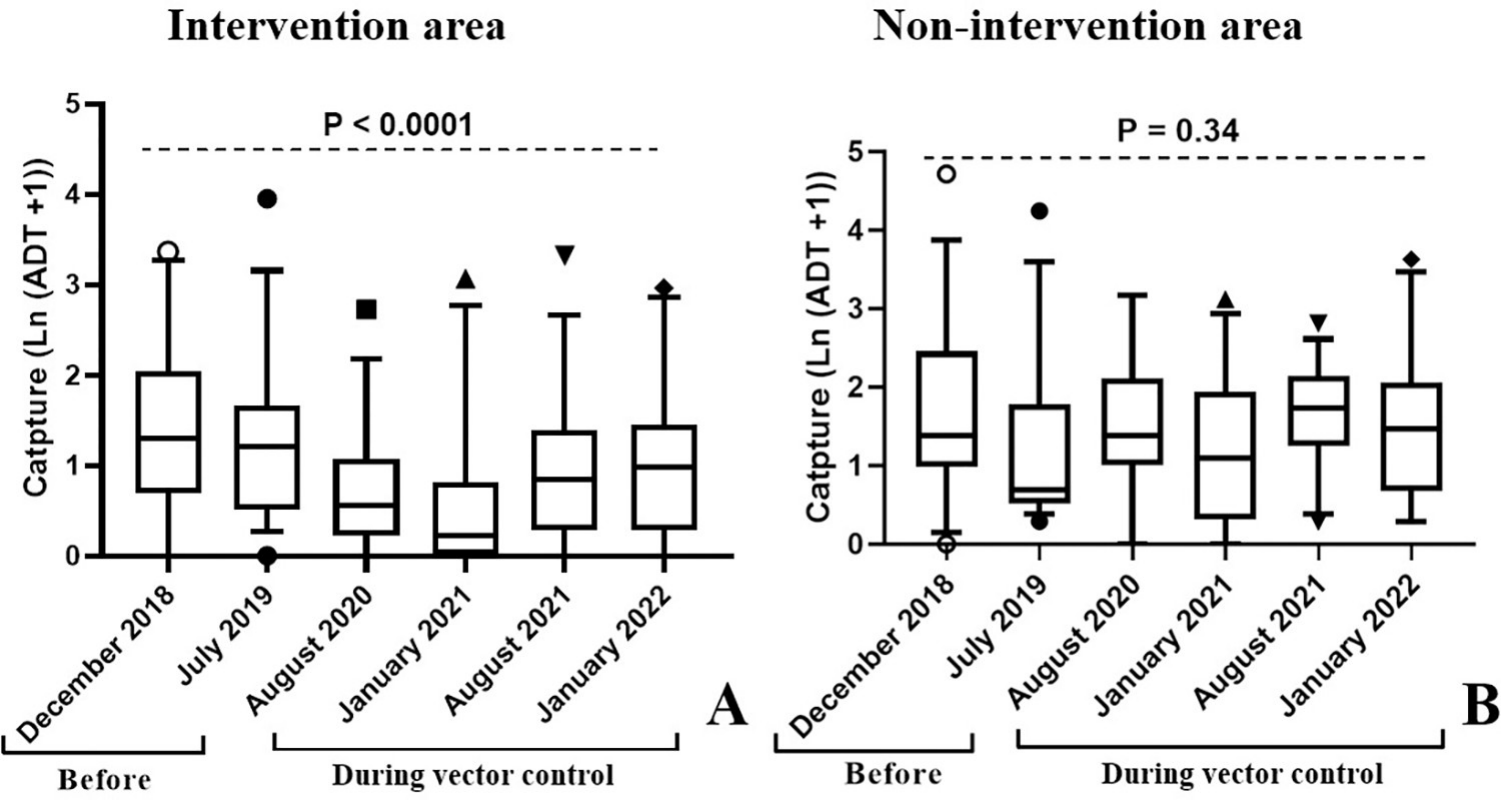
Boxplot of *Glossina palpalis palpalis* apparent densities per trap during the vector control operation.

Post-intervention surveys showed a great reduction of tsetse catches in the intervention area in the first twelve months of vector control, followed by a partial recovery in the next twelve other months. The overall *Glossina palpalis palpalis* density decreased from 2.48 [1.92–3.14] tsetse/trap/day in July 2019 to 0.95 [0.69–1.35] tsetse/trap/day in August 2020, showing significant reduction of 62.50% (p < 0.0001). During the second evaluation after twelve months of vector control (January 2021), the mean catches continued decreasing to 0.66 [0.42–0.94] tsetse/trap/day, for an overall reduction of 73.39%. During the third and the fourth surveys, we observed a partial recovery in tsetse densities from 0.66 in January 2021 to 1.45 [1.07–1.90] tsetse/trap/day in August 2021, and 1.71 [1.27–2.24] tsetse/trap/day in January 2022 (Table 1; Fig 3A).

#### Evolution of tsetse densities in the non-intervention area

A total of 928 tsetse flies were caught in the non-intervention area before the first target deployment, of which 603 were trapped during the heavy dry season (December 2018) and 325 during the light dry season (July 2019). As in the intervention area, four tsetse fly species or sub-species were identified, namely *G*. *palpalis palpalis* with relative abundances 94.68% and 91.69%, *G*. *pallicera* (3.32% and 6.77%), *G*. *caliginea* (1.49% and 0.92%), and *G*. *nigrofusca* (0.50% and 0.62%) for heavy and light dry seasons, respectively (Table 2). *G*. *palpalis palpalis* displayed high densities in the different sampling points in the non-intervention area (Fig 3B), with mean ADT values of 3.98 95%CI [1.48–8.09], and 2.43, 95%CI [0.73–5.77] flies/trap/day in heavy and light dry seasons respectively.

**Table 2:** Summary of the number of flies captured in non-intervention area.

Tsetse species	*G*. *palpalis palpalis*	*G*. *caliginea*	*G*. *nigrofusca*	*G*. *pallicera*	Non identified	Total	*G*.*p*.*p*. ADT
**December 2018**	571 (94.68)	9 (1.49)	3 (0.50)	20 (3.32)	0 (0)	603	3.98 [1.48–8.09]
**July** **2019**	298 (91.69)	3 (0.92)	2 (0.62)	22 (6.77)	0 (0)	325	2.43 [0.73–5.77]
**August** **2020**	314 (95.73)	8 (2.44)	2 (0.61)	4 (1.22)	0 (0)	328	3.74 [1.73–7.21)
**January** **2021**	219 (89.02)	14 (5.69)	6 (2.44)	7 (2.85)	0 (0)	246	2.40 [0.87–5.19]
**August** **2021**	214 (86.64)	10 (4.05)	1 (0.40)	21 (8.50)	1 (0.40)	247	4.13 [2.31–6.96]
**January** **2022**	258 (91.81)	10 (3.56)	2 (0.71)	11 (3.91)	0 (0)	281	3.64 [1.47–7.70]

*G*.*p*.*p*.: *Glossina palpalis palpalis*; Numbers in brackets are relative abundances of each tsetse taxon.

Although *G*. *palpalis palpalis* densities varied in the non-intervention area, it generally increased by 33.24%, i.e., from 2.43 [0.73–5.77] tsetse/trap/day in July 2019 to 3.64 [1.47–7.70] tsetse/trap/day over the 2 years of the study (Fig 3B).

The other tsetse species also present were captured during the 2 years of the study, at almost constant densities (Table 2).

### Impact of tiny targets on trypanosome prevalence in *Glossina palpalis palpalis*

#### Trypanosome prevalence in *G*. *palpalis palpalis* in the intervention area

From 713 and 844 randomly selected flies of the sub-species *G*. *palpalis palpalis* captured in December 2018 and July 2019 pre-intervention surveys, we identified a total of 162 (22.72%) and 166 (19.67%) flies infected with at least one trypanosome species in their bodies, respectively (Table 3). The most frequent species was *Trypanosoma congolense*, with infection rates of 20.90% and 11.97%, followed by *T*. *brucei* s. l. (1.96% and 7.23%), *T*. *vivax* (0.47% and 2.37%) and *T*. *simiae* (0.47% and 1.90%) for the heavy and light dry seasons, respectively. Mixed infections occurred in 11 (1.54%) and 16 (1.90%) flies for the two seasons, and 36 flies (22.22%) from heavy dry season (December 2018) carried mature infections of *T*. *congolense*, with trypanosome DNA being detected in their mouthparts. *T*. *brucei gambiense*, the parasite responsible for human sleeping sickness, were identified in 4 flies among the *T*. *brucei* s. l. infections.

**Table 3:** *Trypanosoma* sp. infection rates before and during the vector control in the intervention area.

Date	Number analized	TC (%)	TB (%)	TV (%)	TS (%)	Mixed infections	Total	95% CI in %
**December 2018**	713	149 (20.90)	14 (1.96)	5 (0.70)	5 (0.70)	11 (1.54)	162 (22.72)	[19.8–25.94]
**July** **2019**	844	101 (11.97)	61 (7.23)	20 (2.37)	1 (0.12)	16 (1.90)	166 (19.67)	[17.13–22.49]
**August 2020**	337	19 (5.64)	47 (13.95)	2 (0.59)	2 (0.59)	2 (0.59)	68 (20.18)	|16.24–24.79]
**January 2021**	297	8 (2.69)	1 (0.34)	1 (0.34)	0 (0)	1 (0.34)	9 (3.03)	[1.6–5.6]
**August 2021**	366	5 (1.37)	2 (0.55)	0 (0)	0 (0)	0 (0)	7 (1.91)	[0.93–3.89]
**January 2022**	395	17 (4.30)	3 (0.76)	1 (0.25)	0 (0)	1 (0.25)	20 (5.06)	[3.48–8.09]

TC: *Trypanosoma congolense*; TV: *Trypanosoma vivax;* TB: *Trypanosoma brucei* s. l.; Tbg: *Trypanosoma brucei gambiense*. In brackets are the flies’ infection rates (percentages).

**Fig 4:**
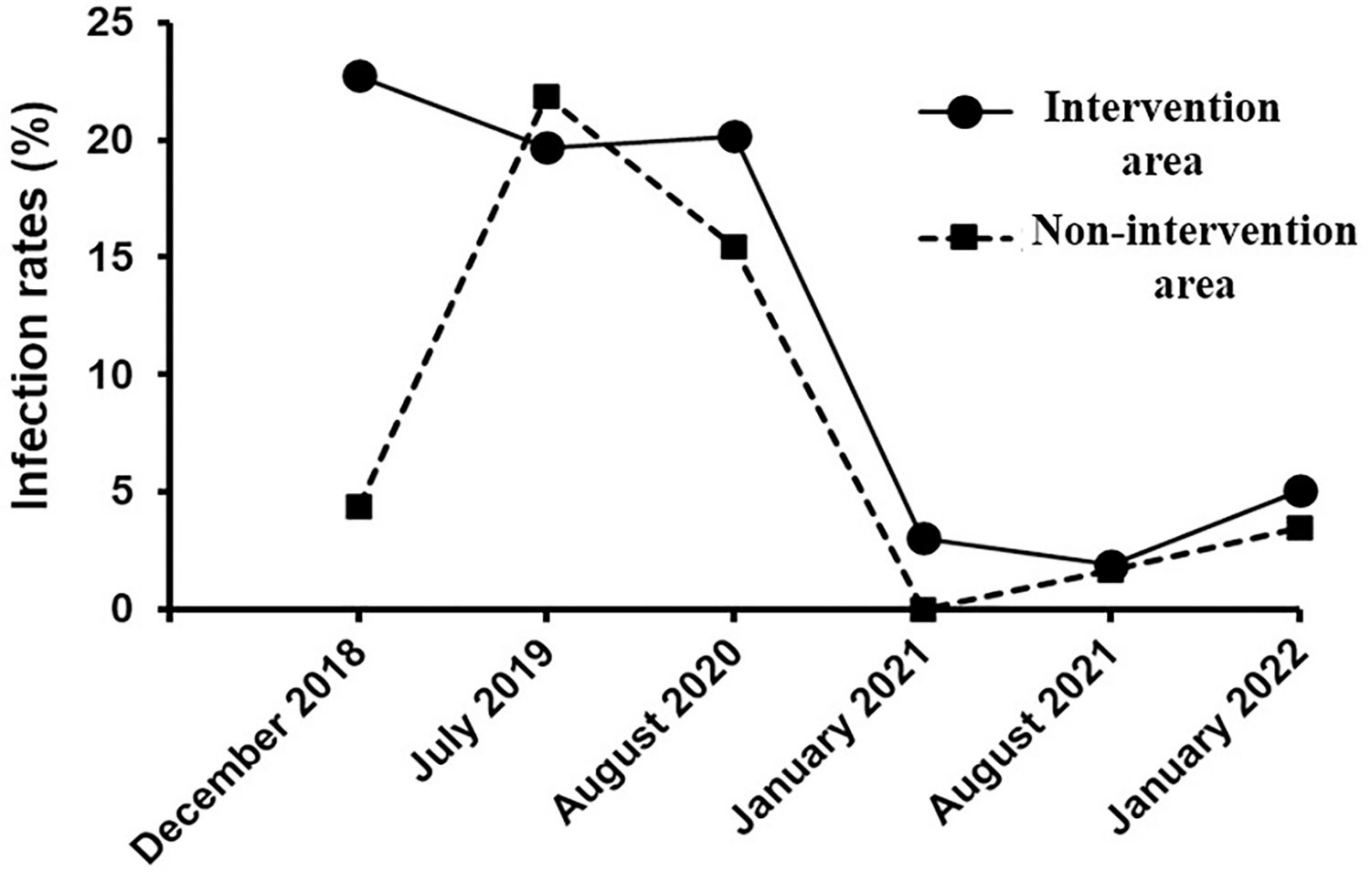
Patterns of trypanosome infection rates in *Glossina palpalis palpalis* in intervention and non-intervention areas.

After the deployment of Tiny Targets, tsetse infection rates remained high after the first six months (from 19.67% in July 2019 to 20.18% in August 2020), but a significant drop was observed at the eighteenth month, with only 3.03% (p < 0.001) of flies being found infected in January 2021 (Fig 4). During the last six months of vector control, tsetse infection rates increased to 5.06%. However, no mature infection was identified in all those infected flies during the intervention.

Vector control equally affected the prevalence of Trypanosome species previously detected; indeed, infection rates of the predominant species *T*. *congolense* decreased from 11.97% before vector control (July 2019) to 4.30% after 2 years (January 2022), *T*. *brucei* s. l. from 7.23% to 0.41%, *T*. *vivax* from 2.37% to 0.25%, and *T*. *simiae* from 1.90% to 0% (Table 3).

### Trypanosome prevalence in *G*. *palpalis palpalis* in the non-intervention area

From 341 and 288 randomly selected *G*. *palpalis palpalis* captured in December 2018 and July 2019 pre-intervention surveys, we identified a total of 15 (4.40%) and 63 (21.98%) flies harbouring at least one trypanosome species in their midguts respectively (Table 4). The most frequent species was *T*. *congolense* with infection rates of 3.81% and 12.50%, followed by *T*. *brucei* s. l. which accounted for 0.59% and 8.33% for the heavy and light dry seasons respectively. *T*. *vivax* and *T*. *simiae* were found only in the light dry season where they accounted for 3.13% and 0.35% respectively. Amongst these infected flies, 7 (2.43%) were carrying mixed infections and no mature infections were recorded.

**Table 4:** Trypanosome infection rates before and during the target intervention in the control area.

Date	Number analized	TC (%)	TB (%)	TV (%)	TS (%)	Mixed infections (%)	Total (%)	95% CI (%)
**December** **2018**	341	13 (3.81)	2 (0.59)	0 (0)	0 (0)	0 (0)	15 (4.40)	[2.68–7.13]
**July** **2019**	288	36 (12.50)	24 (8.33)	9 (3.13)	1 (0.35)	7 (2.43)	63 (21.88)	[17.49–27.01]
**Aug** **2020**	181	2 (1.10)	24 (13.26)	0 (0)	2 (1.10)	0 (0)	28 (15.47)	[10.99–21.57]
**January** **2021**	103	0 (0)	0 (0)	0 (0)	0 (0)	0 (0)	0 (0)	-
**August** **2021**	60	1 (1.67)	0 (0)	0 (0)	0 (0)	0 (0)	1 (1.67)	[0.3–8.86]
**January** **2022**	116	5 (4.31)	0 (0)	0 (0)	0 (0)	1 (0.86)	4 (3.45)	[1.35–8.53]

TC: *Trypanosoma congolense*; TV: *Trypanosoma vivax;* TB: *Trypanosoma brucei* s. l.; Tbg: *Trypanosoma brucei gambiense*. Numbers in brackets are the flies’ infection rates.

After Tiny Targets deployment, there was a significant decrease in the fly’s infection rates from a mean of 21.88% in July 2019 to 3.45% after 2 years of intervention (Fig 4), showing a reduction of 84.23% (p < 0.001). More specifically, infection rates showed significant decrease from 21.88% in July 2019 to 15.47% in August 2020, followed by a drastic decrease to 0% in January 2021. However, we observed an increase to 1.67% in August 2021 and to 5.45% in January 2022 (Table 4).

Considering trypanosome species, *T*. *congolense* decreased from a rate of 15.50% in July 2019 to 1.67% after 2 years (January 2022), showing a total reduction of about 89.22% (p < 0.001). Regarding other species, *T*. *brucei* s. l. infection rate reduced from 8.33% to 0% in January 2022, showing reduction of about 100% (p < 0.001). *T*. *vivax* and *T*. *simiae* rate passed from 3.13% and 0.35% to 0% respectively.

## Discussion

Active case detection and treatment have helped in reducing the sleeping sickness incidence to a low level [28]. Additionally, the introduction of vector control especially with the development of Tiny Targets contributed to curb the incidence still further [8,20]. This tool has proven very important in settings where the disease is sustained for decades at low levels, and where the detection of individual cases could be very expensive. This is the case of Campo in the South Region of Cameroon. The WHO acknowledges the potential benefit of incorporating vector control in the fight against *gambiense* sleeping sickness [29]. Here, we discuss the impact of vector control using tiny targets on tsetse densities and trypanosomes circulating in the Campo HAT focus.

### Evaluation of the impact of targets on tsetse densities

During the tsetse control initiative in Campo using tiny targets (January 2020 to January 2022), the mean daily catches of *G*. *p*. *palpalis* declined by 73.69% in the twelfth month of vector control. This reduction was low, compared to 80% to 90% during the first six months as observed in DCR [30], Chad [31], Côte d’Ivoire [32] and Uganda [11]. One factor that could explain the difference in the pattern of tsetse densities in Cameroon compared to other countries during the vector control could be the marked difference in tsetse habitats between countries. Campo is a dense forest area with high land coverage with vegetation, swampy areas, marshes, and mangroves, representing probably one of the most challenging environments for vector control, as large areas infested with tsetse flies are not easily accessible. Although Tiny Targets were easily installed in most of the mangrove along the Ntem River, most of the small tributaries coming from the mainland and crossing large surfaces of marshes and swamps that seem to be tsetse breeding hotspots were not accessible because of the vegetation congestion on riverbeds. Streams forming those tributaries on the mainland, on which villagers carry out activities were previously shown to harbour high tsetse fly densities [18,19] and we hypothesize that despite the presence of Tiny Targets in accessible areas, there is a constant supply of flies from downstream. Secondly, although more than 90% of tiny targets installed were still in good physical condition (upright position) after six months (that was quite good compared to the 50% expected to have fallen [33]), many were already hidden with vegetation after 3 months or others became less effective because of film of particles deposited on them and preventing flies from getting into contact with the insecticide. This certainly favours slight increase in densities during three months before monitoring, but this hypothesis couldn’t be assessed as in many studies where monitoring is usually done every three and not six months as was the case in our study [8,11,30]. Another hypothesis is that the area covered by the vector control intervention was not sufficient to sustain the decrease in tsetse density and therefore, recolonisation occurred from neighbouring areas not covered by Tiny Targets (i.e., from the Guinean Bank of the river Ntem, following human or animals crossing the border), following a density-dependant process [34–36].

Our study also showed a partial gradual recovery (almost 20%) of the tsetse population density, during the second year of vector control. This result could be explained by environmental mutations or changes that occurred in Campo biotope during the intervention, with the implementation of a new palm grove (3,500 hectares set on 70,000 hectares expected) through a huge deforestation programme near the Campo natural game reserve. Indeed, the increase in tsetse densities in the second year coincided with villagers’ complaints of elephants invading the villages, destroying crops, and these elephants are known locally to move along with masses of tsetse flies. As deforestation was done without preliminary orientation of animals to the natural game reserve side, the animals that were on the villages side probably came close to villages to avoid the noise, and unfortunately constituted permanent tsetse attractants.

In the non-intervention area as expected, tsetse densities stayed high over the two years of monitoring, with a slight non-significant increase in the second year, also following elephants coming near villages since the start of deforestation for the palm grove implantation. This result confirmed that tiny targets were the factors responsible of reducing the tsetse densities in the intervention area.

### Evaluation of the impact of targets on trypanosomes circulating in tsetse flies

This study showed a high reduction in the prevalence of trypanosomes in tsetse flies captured in Campo during the vector control. Overall, trypanosome infection rates decreased by 95% in the intervention area during the first 18 months of vector control. However, a decrease of trypanosome infection rates was also observed in the non-intervention area. This finding indicates that the non-intervention area was not far enough from the intervention area and that the same parasite populations circulate in the whole Campo focus through animal movements. The impact of vector control on trypanosome circulation was higher than that recently reported in Côte d’Ivoire [37], where tsetse infection rates were generally lower following the deployment of targets but not significantly, despite a great reduction in tsetse densities. Furthermore, in this study, no *T*. *brucei gambiense* infections were detected amongst all flies captured during the two years of vector control; this result indicates that the circulation of the human parasite has possibly regressed in the area. More importantly, no mature infections were detected in flies captured during the vector control, indicating that the infections detected in tsetse were recent, and flies infected were likely to get into contact with Tiny Targets and die before maturing and transmitting trypanosomes to humans or animals.

## Conclusion

This study has shown the positive impact of the Tiny Targets in reducing the tsetse vector population density in Campo that could be associated to a good reduction of human and animal trypanosomes circulation. This tool can help in achieving the elimination of sleeping sickness in Cameroon. However, the impact of vector control was not to the expected magnitude, when compared to the levels achieved in other countries. Integrated action involving the actors of the palm plantation under development is required to mitigate the effects of the plantation on vector control. Our results also suggest that increasing the targets coverage to the non-intervention area and to the Guinean banks of the river Ntem in Rio Campo, could help minimize levels of migration and its impact. Maintaining the targets deployed every 3 months through clearing around and changing the ones recovered with particles may also improve efficiency of the vector control.
